# Struggling to survive for the sake of the unborn baby: a grounded theory model of exposure to intimate partner violence during pregnancy

**DOI:** 10.1186/1471-2393-14-293

**Published:** 2014-08-28

**Authors:** Hafrún Finnbogadóttir, Anna-Karin Dykes, Christine Wann-Hansson

**Affiliations:** Department of Care Science, Faculty of Health and Society, Malmö University, Malmö, Sweden; Department of Health Sciences, Medical Faculty, Lund University, Lund, Sweden; The Swedish Institute of Health Sciences (Vårdalinstitutet), Department of Health Sciences, Lund University, Lund, Sweden

**Keywords:** Intimate partner violence, Pregnancy, Experience

## Abstract

**Background:**

Intimate partner violence (IPV) during pregnancy is a serious matter which threatens maternal and fetal health. The aim of this study was to develop a grounded theoretical model of women’s experience of IPV during pregnancy and how they handle their situation.

**Method:**

Ten interviews with women who had experience of being exposed to IPV during pregnancy were analyzed using the grounded theory approach.

**Results:**

The core category ‘Struggling to survive for the sake of the unborn baby’ emerged as the main concern of women who are exposed to IPV during pregnancy. The core category also demonstrates how the survivors handle their situation. Also, three sub-core categories emerged, ‘Trapped in the situation’ demonstrates how the pregnant women feel when trapped in the relationship and cannot find their way out. ‘Exposed to mastery’ demonstrates the destructive togetherness whereby the perpetrator’s behavior jeopardizes the safety of the woman and the unborn child. ‘Degradation process’ demonstrates the survivor’s experience of gradual degradation as a result of the relationship with the perpetrator. All are properties of the core category and part of the theoretical model.

**Conclusion:**

The theoretical model “Struggling to survive for the sake of the unborn baby” highlights survival as the pregnant women’s main concern and explains their strategies for dealing with experiences of violence during pregnancy. The findings may provide a deeper understanding of this complex matter for midwives and other health care providers. Further, the theoretical model can provide a basis for the development and implementation of prevention and intervention programs that meet the individual woman’s needs.

## Background

Violence against women is a well-known public health problem worldwide, and it is also a violation of human rights [1]. The period of pregnancy is unfortunately no protection against intimate partner violence (IPV) [2, 3]. Previous Scandinavian qualitative studies highlight the complexity of being pregnant and abused by the intimate partner [2, 3] and emphasize the woman’s need for “keeping up a front” for the surroundings [3] due to difficult existential choices related to ambivalence [2]. Women who are afraid of their intimate partner both before and during pregnancy have poorer physical and psychological health during pregnancy [4, 5]. Thus, IPV during pregnancy is a serious matter that threatens maternal and fetal health outcomes [1, 6–10].

Worldwide, the prevalence of violence against pregnant women ranges between 1.2 – 66% [6]. A WHO study showed that between 4-12% of women are subjected to physical violence during pregnancy, and in more than 90% of the cases the perpetrator is the biological father to the unborn baby [11]. In Swedish studies the prevalence of physical or sexual abuse during pregnancy varies from 1.3% to 11% [12–14]. This variation is probably attributable to the use of different methods, definitions, and cultural differences, thus making it difficult to compare results across studies [15]. The prevalence of IPV may also be underreported because of shame and fear of escalation of the abuse should the abuse become known [3, 15, 16]. IPV during pregnancy is defined in this study as physical, sexual or psychological, mental or emotional violence, or threats of physical or sexual violence that are inflicted on a pregnant woman by an intimate male partner, or marital/cohabiting partner. This definition has been modified from recommended definitions by Krantz and Garcia-Moreno [17].

Exposure to violence during pregnancy is often unrecognized and/or unsuspected by others, and therefore not addressed by professionals in health care settings [9]. A Swedish interview study with midwives working in antenatal care (ANC) highlighted the vulnerability of the unborn child and the need to provide protection by means of adequate care to the pregnant woman living in a violent relationship [18]. The midwives themselves and their own personal barriers may be the main obstacle to working with this delicate matter, and therefore it is necessary to provide carefully designed educational programs to all clinically active midwives [18]. However, lack of consensus exists as to whether routine screening of domestic violence during pregnancy can be justified, thus illustrating the complexity of this controversial subject. A Cochrane review shows that screening for women exposed to IPV in health care settings is likely to increase detection rates, but evidence is still lacking concerning the long-term benefits for the violence-exposed women. Further, no study has compared the benefits of universal screening versus selective screening for high risk groups, such as pregnant women [19]. Another Cochrane review showed insufficient evidence regarding the effectiveness of interventions for domestic violence in relation to pregnancy outcomes [20]. Although evidence-based interventions are needed, little is known about the actual experiences and primary concerns of women exposed to violence during pregnancy. In order to gain a deeper understanding regarding the subjective experience of exposure to violence during pregnancy, it is necessary to develop a theoretical model that reflects the survivors’ behavior and needs. The aim of this study was to develop a grounded theoretical model of women’s experiences of intimate partner violence during pregnancy and how they manage their situation.

## Method

The grounded theory method, as developed by Glaser [21, 22], was considered suitable for the aim of the study. The research questions were: *What are the women’s experiences of being exposed to violence during pregnancy? What are the emerging concepts described by the women?* In grounded theory it is behaviors, not individuals, which are categorized [21]. The grounded theory method is used to build a theoretical model of what is happening and how the situation is handled [21]. In the present study, the patterns of behavior are those described by women who have experienced intimate partner violence while pregnant.

### Settings and participants

Women were eligible for inclusion in the study if they were mothers living in the Scania region in Sweden, had experience of being exposed to IPV during pregnancy (survivors), were separated from the perpetrator, and able to speak and understand Swedish. Ten women aged 21–44 years agreed to participate in the study. Their educational level ranged from less than high school up to university studies. Eight women were Swedish-born, among whom two had foreign-born parents and two were immigrants. Eight women had only one child with the perpetrator and were primiparae. Two were multiparae and had three, respectively, two children with the perpetrator. The duration of the relationship with the perpetrator varied from 1.5 to 20 years. The age of the woman’s youngest child ranged from 5 months to 4 years.

### Data collection

The data collection was performed between December 2011 and May 2012. Recruitment of participants ended when no new information was forthcoming, indicating that saturation had been achieved. Eight women were recruited by two welfare officers working at women’s shelters who acted as gatekeepers. They informed all their clients who fulfilled the inclusion criteria about the research project, showed them an announcement about the study and inquired about participation. All women agreed to participate, and either the welfare officer acted as an intermediary or the survivor contacted the main researcher by herself. Two women responded to announcements that had been posted at two separate emergency wards for women and contacted the first author (HF). The informants received written information about the study before they made their decision, and they were given the opportunity to obtain further clarification from the first author. The informants voluntarily gave their written consent to participate and spoke freely about their lived experience, through narratives, of intimate partner violence before and during pregnancy. All interviews began with informal talk about the child/children and questions about the women’s background (age, education, etc.), following which the main research question was posed: *Will you please tell me your story, your experience of being exposed to violence during pregnancy?* The question was often followed by some explanation that such violence could be both physical and psychological. More specific questions were posed later during the interview, such as *how did you manage?* The first author conducted all the interviews. The women were interviewed in a safe place of their own choosing, so that they could feel free to talk at their own convenience. Five interviews were performed at the informants’ homes, three at the women’s shelter and two at the university. The interviews lasted between 49 minutes to 3 hours and 20 minutes.

### Analysis

The inductive analytic process started already during the interviews, and the first author also listened to the recorded text shortly after each interview and memos were written down. During the data collection period the first author used a notebook where memos, thoughts and ideas were written down. In the grounded theory concept “all is data” p.12 [21]. The data collection ended when saturation in the categorization was reached. The open coding started immediately in connection with the transcription of the interviews, performed by the first author (HF). Also, the two co-authors independently carried out open coding of two randomly chosen interviews. Afterwards, the authors compared and discussed their coding results, and consensus was reached. The NVivo program was used for gathering and grouping data. The substantive coding of the material continued, and memos and annotations were continually created. During the coding process the following questions were considered: *What is this data, and how does it fit into the study? What category does this incident indicate? What is actually happening in the data? What is the informant’s main concern? How does the informant deal with this concern, and how is the concern resolved during the pregnancy?* Constant comparison of incidents generated categories and their properties. Already in the first interview a conceivable core category emerged. When a mutual decision was reached designating this as the core category, the selective coding process started, i.e. which meant coding solely material that related to the core category and its concepts or property. The theoretical memos, illustrated by figures and written text, were discussed throughout the entire analytic process. When saturation was reached regarding the core category and its concepts, the next stage of the analysis was to identify the emerging theoretical codes such that the underlying patterns became visible and could be aggregated into a theoretical model. According to the grounded theory method, a literature review was not carried out until the theoretical model had emerged. We confirm that, the guidelines for Qualitative research review (RATS) as outlined and modified for BioMed Central has been followed.

In accordance with Krantz and Garcia-Moreno [15], the following definitions of violence were utilized during the analytic process: *Physical violence* is exercised through physically aggressive acts such as kicking, biting, slapping, and beating or even strangling. *Psychological, mental, or emotional violence* describes acts such as preventing a woman from seeing family and friends, ongoing belittlement or humiliation, economic restrictions, violence or threats against cherished objects and other forms of controlling behaviors. *Sexual violence* includes forced sex through the use of physical force, threats, and intimidation, forced participation in degrading sexual acts as well as acts such as denial of the right to use contraceptives or to adopt measures to protect against sexually transmitted diseases [15].

### Ethical considerations

The informants were given written and verbal information about the aim of the study and the nature of the interview, and were informed that they could end the interview at any time. Furthermore, all women were informed that their participation in the study was anonymous, that all information would be treated with confidentiality, and that the presentation of the findings would ensure that individuals could not be identified. After the interview the first author made sure that the informants were not psychologically distressed due to the interview and that there was no need of immediate emotional support. According to the Declaration of Helsinki [23] the likelihood of benefits from the current research was considered. Violence during pregnancy is a research topic that raises important ethical and methodological challenges in addition to those challenges that are related to research on human subjects in general [24]. The World Health Organisation’s (WHO) ethical and safety recommendations for research on domestic violence against women have therefore been followed [23]. Approval for the study was provided by the Swedish Regional Ethical Review Board (Dnr: 2011/336, 2011/703).

## Results

**Figure 1:**
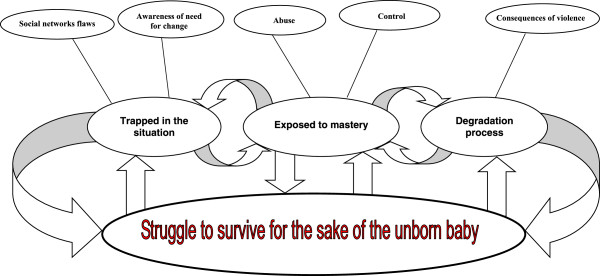
**A theoretical model “Struggle to survive for the sake of the unborn baby”.**

The core category, ‘Struggling to survive for the sake of the unborn baby’ and three sub-core categories, i.e. ‘Trapped in the situation’, ‘Exposed to mastery’ and ‘Degradation process’, together with five categories, emerged from the data and formed the theoretical model (Figure 1).

The analysis revealed that women who experienced IPV during their pregnancy were deeply concerned not to harm the unborn baby. Their main concern emerging from the interviews is *‘Struggling to survive for the sake of the unborn baby’.* The survivors deal with constant fear and violence during their pregnancy and are emotionally overloaded. They worry about whether the noise and abuse they are exposed to can affect the pregnancy and the unborn baby. The entire pregnancy revolves around not making ‘that person’ upset or mad and to survive despite the perpetrator’s impulsive anger. A deliberate choice is to stay in the relationship despite the abuse, and to avoid exposing the unborn baby to the additional stress that might be provoked by divorce proceedings, custody and support issues, etc. Stress is considered by these women to be more dangerous for the unborn baby, and they regard stress as increasing the risk for premature birth. *“I worried very much that I might cause her harm because I had this inner stress… I had this inner stress all the time, the whole time, and I was terrified that it was going to damage her”* (I:7).

The interaction between the couple triggers the violence and the pregnancy is tinged with constant brawl and violation. The women’s feelings of joy about the pregnancy are replaced by terror and fear. The survivors expressed anxiety regarding their unborn baby’s health and handled the situation in different ways according to the complexity of the situations. The survivors expressed caring for their unborn baby and attempting to minimize the effect of the abuse as much as they could by means of different coping strategies. For example, a survivor could in a dialogue with herself realize that she needs to protect the unborn baby, thereby convincing herself that the relationship will become better postpartum. Worries about whether the unborn child would be affected by the mother-to-bee’s sadness were common among these women. The mother-to-be tries to avert her thoughts by walking, reading books and watching TV. The survivor also copes by talking to the ‘belly’ and creates a relation with the unborn baby. She convinces herself and the unborn baby that together they can carry it through. Sometimes the survivors make an effort to answer back and to stand up for themselves. However, their awareness of the pregnancy and the life growing inside forces them to resign themselves to their situations and not to take any risks that might lead to an escalation of the violence. Although a woman can feel so depressed that she considers taking her own life, the unborn baby’s existence prevents such acting. Step by step the survivors adapted to the perpetrator to avoid brawls, fights and insults, because they sought to protect the unborn baby. *“He leaned over the table and started to hyperventilate. I managed just in time to leap to the side so the table flew right into the wall… I was so scared. He started to scream and howl …. I am crying and I can only think about my belly”* (I:6)*.*

### Trapped in the situation

The women felt *“trapped in the situation”* i.e. pregnant and exposed to violence by their partner and sometimes also exposed to violence by another family member in his family (domestic violence). ‘*Trapped in the situation’* is a property of *“struggle to survive for the sake of the unborn baby”* and demonstrates how the women felt when trapped in the relationship. All of the relationships started out with the idea of romantic togetherness. Some women made a commitment directly from a stable relationship (experienced as dull), throwing themselves into the “storm” of a new relationship. Metaphorically, the pendulum swung completely over to the other side. Also, some women were very much influenced by perceptions concerning commonalities with regard to spirituality and culture. Nevertheless, initially the women were voluntarily trapped. Early in the relationship there may have been some warning signals, such as spurts of anger and controlling behavior, but the survivors did not want to hear or see these signs and were prone initially to interpret these as attention and caring. The man was experienced as very decent, fun, and devoted. However, such behavior tended to last only as far as the relationship remained the way he wanted it to be. The love affair either led to pregnancy very early in the relationship or after many years of confinement. The women felt trapped in the pregnancy and they expressed their love for the new unborn life in unconditional terms. *“Of course, my future was lying there inside me, so …… I felt like I couldn’t go on because I felt so awful…. But I had no choice. You can’t just say, I have had enough, really, because I am the one carrying this responsibility, …. I can’t do anything except try to keep going on”* (I:4)*.* The survivors also believed that the relationship would become better because of the pregnancy and they looked forward to the possibility of family happiness.

The category *‘Awareness of need for change’* is a property of the sub-core category *‘trapped in the situation’* and demonstrates how the survivors became aware of their complex situation, i.e. to be pregnant and abused by the man they had fallen in love with. Some made attempts to seek help with the situation and others did not seek help due to shame. For example, the healthcare givers were told by one survivor that her husband was not acting decently towards her. No initiative from the staff was evident, even though they were listening. In one case, a woman also told her ANC midwife everything, since she had decided to divorce her husband. However, ultimately she had no strength to divorce him in her condition and was ‘*trapped in the situation’.* Another woman called her mother and told her about her difficult relationship and that she did not have the energy to live in this situation. She wanted to finish the relationship and to leave her tormentor for good. The mother expressed sympathy with her daughter’s difficulties, yet she said *“Can’t you stay with him anyway… so she didn’t support me completely”* (I:2)*.*

Lack of societal resources contributed to the women’s decision to remain in the abusive relationship. The category ‘social network flaws’ is therefore also a property of the sub-core category ‘Trapped in the situation’. Regular time with a welfare officer might have at least helped a woman to air the pressure she had at home. However, these women’s lives were characterized by social isolation and control. The woman’s daily life shrank when the perpetrator never allowed the woman to meet friends or parents by herself. The women struggled to get the perpetrators to change and to improve themselves, all to protect the unborn baby. However, all promises regarding change were only empty words. Before the women could really become aware of what was happening to them, they became metaphorically ‘trapped in the tornados’ and could no longer control the situation and find their way out. These survivors lived in solitary confinement and in a false scenario, longing for family happiness. Courageous attempts to fight back to regain control worsened their situation with increased assaults, leading in turn to even more feelings of entrapment in a difficult situation.

### Exposed to mastery

The pregnant women were “*exposed to mastery”* by the perpetrator and they needed to shield themselves and the unborn baby. ‘*Exposed to mastery’* is a property of *‘Struggling to survive for the sake of the unborn baby’* and demonstrates the destructive togetherness where the perpetrator is very dominating and uses psychological and mostly also physical violence to get his will. The relationship is only on his terms. The women’s stories, which reflect their memory of the perpetrators’ behavior while pregnant, contained descriptions of exposure to *psychological* inclusive *economic violence* and *physical* inclusive *sexual violence*. The perpetrators’ behavior jeopardized the family unit and the safety of the woman and their unborn child.

Women who had earlier experience of abuse primarily tried to adapt to avoid flare-ups, as a means of protecting the unborn baby. However, women who were experiencing the abuse for the first time during their first pregnancy initially fought back verbally and physically until they realized that they might hurt the unborn baby, and then they became resigned. The perpetrator controlled every step the woman took and demanded that she report everything she did, as in a cross-examination. *“So I couldn’t dispose over my own time like I would have wanted to”* (I:6)*.* Bit by bit the survivor had to erase both friends and family from her life. Email, face book and mobile phones were controlled. Gradually her life world shrinks and she becomes socially isolated and struggles to survive on her own. At the same time as the belly became bigger, the flare-ups occurred more frequently and the violence escalated to another level. The perpetrator could be manipulative and become charming when necessary. A typical maneuver was for the perpetrator to express regret with flowers or presents, as if he were afraid that the woman would abandon him. Every day was characterized by threats and criticism and often with fighting and tears. “*I just felt that my life was total darkness; I felt that I was truly in hell; that was the way I felt when I was with him”* (I:6)*.*

Sometimes the perpetrator alternated between *“*cold and hot*”,* i.e. when the woman was broken down, he consoled her and in that way he felt “big and strong” (as expressed by one of the survivors). The psychological violence could also appear as indifference towards the pregnancy, or inattentiveness with regard to the pregnancy or the changes in the woman’s body due to the pregnancy. For example, one woman experienced no empathy and was left alone at the delivery ward, bleeding in the early part of the third trimester, and it was not until three days later that her boyfriend returned. *“I didn’t have a cell phone with me because it was acute, so I gave birth to a baby … alone… and I didn’t know what was going on”* (I:4)*.*

The physical intimacy disappeared as the pregnancy advanced and the perpetrator could also have love affairs with other women. Economic violence could take the form of gambling away the woman’s entire savings or as cheapness regarding the woman’s every day needs. The threats escalated as the pregnancy advanced. Threats such as knocking out her teeth or death threats exacerbated the level of psychological violence until the survivor became very stressed and petrified of her tormentor. *“Now he said I’m going to kill you…. And he got that dark evil look and he trembled and hyperventilated”* (I:6)*.*

The physical violence also escalated as the pregnancy advanced. In the beginning it could be a slap and a grab, but also a kick in the chest resulting in fracture and both physical and psychological health consequences. Escalation of psychological and physical violence with aggression, hits, hair pulling, spitting on and verbally abusing could occur if the delivery date was overdue. Also, sexual violence occurred in the women’s stories. The perpetrator’s jealousy against the growing belly was obvious; he did not show any respect towards the belly. When they made love, it was the perpetrator that “got sex” and he was very brutal and the woman was often in pain. Several rapes by the perpetrator were experienced and some woman did not dare to move. “*It was better just to give him what he wanted…. I was forced to; maybe I cried and tried to push him away… No”* (I:1)*.*

The perpetrators’ need of power and control dominated the relationships, manifesting itself both in small (what kind of soap the woman uses) and larger matters. The perpetrator was almighty and a woman could be forced to terminate an initiated in vitro fertilization or forced to have an abortion (against her will and beliefs) the first time that she was pregnant. Such experiences were very difficult to live with.

### Degradation process

Gradually the pregnant women became psychologically and physically degraded. *Degradation process* is a property of *“Struggle to survive for the sake of the unborn baby”* and illustrates how the survivors expressed their degradation process as a result of their relationship with the perpetrator. The brawls and fighting made the survivor weaker and weaker as the pregnancy advanced. The women felt that they were drained of energy and exhausted. The survivors’ hope that the perpetrators behavior would change faded away. The last hope could for some women, however, be the birth of the baby. “*I wanted to leave him, ….., maybe the birth would calm him down, when he got the chance to hold the baby in his arms, maybe he would come to realize that … I hoped somehow that he would be overcome with some sort of fantastic feeling of love… the last tiny shred of hope that when he became a father, then he would … if anything was going to calm him down, it would be that”* (I:6)*.*

The survivors who lived in the relationship for many years ultimately were no longer themselves. *“He actually transformed me into somebody I’m not”* (I:3)*.* The survivor’s self-image was twisted and they were filled with blame and shame irrespective of how long the relationship had lasted. “*He poisoned my blood”* (I:9) or *“I felt how he crept under my skin”* (I:6). As the pregnancy advanced, the women’s psychological health became worse and they felt increasingly concerned about the health of the unborn baby.

Lack of sleep was central, and during pregnancy the survivor never obtained sufficient rest because of the constant fights. The perpetrator did not have any empathy or understanding for the pregnant condition and could wake the woman up in the middle of the night to scold her. The constant control and the stress contributed to the degradation. Finally, the easiest way to survive the pregnancy was to give up and to put down the battle-axe. “I just couldn’t deal with the ”battle” so even though it felt wrong, I moved back”. The fights and the insults continued, and the perpetrator gradually eroded the women’s self-esteem. The survivors’ psychological health deteriorated and they became depressed and anxious during the course of the pregnancy. *“I felt very sad during pregnancy, and it is supposed to be a happy time; during pregnancy you are supposed to feel happy, but I didn’t. I feel sad now (she weeps silently) when I talk about it”* (I:5).

## Discussion

In this grounded theory study a core category derived from the empirical data emerged: ‘Struggling to survive for the sake of the unborn baby’ (Figure 1) which means that the main concern for the survivors are to survive the pregnancy for the sake of their unborn baby. Also, it explains how the survivors handled their difficult situation. In practice this knowledge is very important for clinically working midwives and other health care providers. For example, in fact violence–exposed pregnant women are prone to stay in the relationship during pregnancy in order to protect their unborn baby. Also, signs of anxiety, stress and sleeplessness can be indicators of domestic violence. The social behaviors that are demonstrated by the theoretical model do not represent a linear process, but rather a process that moves back and forth between the three sub-core categories, all of which are interrelated. ‘Trapped in the situation’ explains how the pregnant women felt when trapped in the relationship. The initial development of the relationship was included in this sub-core category. The women can’t find the way out of this destructive relationship because of the pregnancy and lack of social support. In practice the midwife can in a natural way be the survivors’ social support during the pregnancy. However, the first step is to disclose the violence. ‘Exposed to mastery’ explained the destructive togetherness whereby the perpetrator’s behavior jeopardizes the safety of the woman and the unborn child. This phase was chronic during the relationship, and the violence increased as the pregnancy advanced. ‘Degradation process’ explained the survivor’s gradual degradation as a result of the relationship with the perpetrator and was connected to ‘Exposed to mastery’ and constantly reiterated in every new situation. However, all three sub-core categories with categories are properties of the core category ‘Struggling to survive for the sake of the unborn baby’ (Figure 1). This model may constitute a basis for the development and implementation of targeted prevention and intervention programs meeting the individual woman’s needs. The current findings highlight the importance of being able to identify those women who are exposed to IPV during pregnancy and the importance of being attentive towards their needs. However, it is not sufficient to be attentive towards the survivor; it is also necessary to have a plan of action [18] and to act depending upon how serious the violence is judged to be.

It should be emphasized that the stories were obtained in a Swedish context by women who recently had left the violent relationship, and some did not at all feel safe whereas others had developed a new life situation. All of the women had received professional support at some point or had talked to a welfare officer. Although some time had passed since their delivery (5 months-4 years), their memories of their pregnancies were fresh in their minds.

The four fundamental sources of validation of a grounded theory (GT) are: fit, relevance, workability and modifiability [21, 22]. A grounded theory model is never right or wrong; such a model only has more or less fit, relevance, workability and modifiability (ibid). The first criteria ‘fit’ refers to how closely the concepts describe the data, the incidents or patterns they are representing. In this case, concepts and patterns that emerged in the empirical data clearly emphasized the women’s concerns when pregnant and exposed to violence. The second criteria ‘relevance’ deals with the emerging concepts of the subjects’ real concern. GT generates a theory about what is actually happening in the data. ‘Struggling to survive for the sake of the unborn baby’ with the three under core-categories appeared clearly in the survivors’ stories. The third criteria ‘workability’ refers to how the concepts are integrated with the theory in terms of the core category and the under core categories. All possible variations of behavior in the studied area were described, including how the women handled the main concern. The present study highlights the complexity and the individual variation of the women’s experiences and also how they handled their situation. The fourth criteria ‘modifiability’ ensures that the theory is not forced onto the data, but rather is modified by it, as in the present study. The literature review gave indications of reasonable relevance, workability and modifiability.

The literature review concerning this topic [2, 3, 25–27] confirmed that the current findings were in accordance with previous studies. However, the core concept ‘Struggling to survive for the sake of the unborn baby’, which emerged as the violence-exposed pregnant woman’s main concern, has not previously been identified. In a Swedish qualitative study [3] the notion of “struggle” is apparent in the survivors’ need for “keeping up a front”. This strategy was employed by violence-exposed women to shut others out while making up their minds about how and when to act and change their lives. In a GT study from the USA [26] concerning abuse during pregnancy the core category was “Living two lives” and referred to the abused pregnant woman’s perception that she was living two different lives. One life was public, reflecting the pregnancy, and the other life reflected the abusive relationship [26]. Further, a later study by Lutz et al. [27] integrated the theory of abuse with the theory of becoming a mother, as a way of understanding women’s behavior and responses to IPV during pregnancy. The concept “struggling to survive” in the final stage of their theory reflected recovering after leaving the abuser and the survivors’ grief and search for meaning. Engnes et al’s [2] qualitative study from Norway highlighted the phenomenon as characterized by ambivalence and difficult existential choices. Additionally, a study [25] that aimed to help providers to better understand the experience of abused pregnant women suggested specific clinical stage-based interventions to assist women at various points in their struggle to survive. All in all, the findings from the earlier studies [2, 3, 25–27] and the present study show that the women not only struggle for their own survival, but as in our study, struggle primarily for the sake of the unborn baby.

In our theoretical model the concept ‘Trapped in the situation’ demonstrates how the pregnant women feels trapped in the marriage, pregnancy and the tornados of violence and cannot find the way out. The concept ‘Trapped in the situation’ has been addressed in Landenburger’s theoretical model, in the second phase (of four), i.e. the ‘enduring phase’, which described the entrapment in and recovery from an abusive relationship in non-pregnant women [28, 29]. Libbus et al. [30] conducted a qualitative study to describe pregnant women’s relationships with abusive intimate partners using Landenburg’s [28, 29] four phase model: binding, enduring, disengaging and recovering as a theoretical framework; however Landenburg’s model appeared not to have a good fit with regard to pregnant women. In Libbus et al’s [30] study the women became trapped and endured violent relationships if they perceived this to be the best strategy for their unborn child. This is in accordance with the core category ‘struggling to survive for the sake of the unborn baby’ in our theoretical model. Our findings seem to extend Landenburg’s [29] theoretical model and also include pregnant women. This is important knowledge for midwives, other health care personal and providers because the violence-exposed pregnant woman needs special support and empowerment during her pregnancy. The survivors remain in the relationship because it feels safer for the unborn baby and possibly safer for the other children in the family as well. Therefore, it is extremely important for the caregiver to show that she/he respects her decision and to give the pregnant woman the necessary information about how society can help.

The concept ‘Exposed to mastery’ demonstrates the survivors’ experience of destructive togetherness filled with both psychological and physical violence. Other researchers [2, 3, 25–31] working with the same topic have not exactly touched upon the concept ‘Exposed to mastery’ in the same manner. The experienced violence is multi-faceted and an individual approach is necessary to meet the unique person’s needs. Relational ethics, which means to be sensitive to a particular situation through opening a dialogue between and among individuals [32], is a very suitable approach to the situation when disclosure of domestic violence occurs. Also person-centered care can be a useful model in these situations [33] which means an attitude of being with people in a respectful and non-hierarchal way. Person-centered approach is a collaborative approach whereby the provider (i.e. midwife) evokes the person’s own intrinsic motivation and resources for change (ibid). However, provision of care needs to be coordinated and integrated to meet the individual needs and health concerns.

The concept ‘Degradation process’ refers to the survivors’ inability to leave the abusive relationship despite their intentions, because the women’s self-esteem and self-respect has faded away and they feel drained of energy. The survivor wants to believe that the violence will come to an end when the baby is born. The feeling of a physically exhausted body and powerlessness was also seen in Engnes et al’s study [2]. Further, Campell and Campell [34] proposed that the pregnant woman most likely stays in the relationship during pregnancy because she wants to make the relationship work, and she believes that having a baby will reduce the abusive behavior. Also, in an Australian study [31] the women’s experiences were variously described as loss of self, being controlled and destruction, aspects which are related to the meaning of the concept ‘Degradation process’ in our theoretical model.

Our main findings showed that women with experience of IPV during pregnancy were deeply concerned about not harming the unborn baby. Moreover, their efforts to find their way out of a severe situation were fraught with difficulties and often were poorly received. Thus, the survivor was often exposed to a “two-faced reaction” on the part of the healthcare personnel. In other words, an authority person such as a doctor, midwife, welfare officer or relatives and friends listened to a violence-exposed woman’s story, but did nothing to help. This can be a sign of lack of knowledge about this delicate matter and may also indicate the need for attitude changes in society. Ultimately, it may be evidence in support of the notion that plans of action at ANC are non-existent, as earlier shown [18]. Midwives are in a unique position to work with this sensitive matter since they have continual contact with the pregnant woman. These women need to have a permissive environment and to be treated with sensitivity and non-judgmental, empathetic behavior. Furthermore, the midwives’ role is not only to identify and support the violence-exposed woman during her pregnancy, but also to refer her to a person with professional expertise within this area. Therefore, it is crucial to have a well- thought-out plan of action, such that healthcare givers know what to do when they encounter a woman who is exposed to violence during pregnancy. However, midwives’ and all health-care personnel’s knowledge also needs to be grounded in the survivor’s own experience.

## Conclusions

The theoretical model “Struggling to survive for the sake of the unborn baby” highlights survival as the pregnant women’s main concern and explains their strategies for dealing with experiences of violence during pregnancy. Such a model may serve as a useful source of information about this complex matter for midwives and other care providers’ and also as a guide to the basic concerns of the violence-exposed pregnant woman. Further, the model can provide a basis for the development and implementation of prevention and intervention programs meeting the individual woman’s needs.

### Clinical recommendations

Survivors of violence during pregnancy need help to navigate among possible services and authorities, and a continuum of professional services in society is essential. Therefore, collaboration between different authorities is crucial and must be smooth and seamless for the violence-exposed (pregnant) women.
